# PERFLUOROALKYL ACIDS: What Is the Evidence Telling Us?

**DOI:** 10.1289/ehp.115-a250

**Published:** 2007-05

**Authors:** Kellyn S. Betts

It was 2000 when the scientific community first became widely aware that perfluorooctanyl sulfonate (PFOS), then the key ingredient in 3M Company’s popular Scotchgard stain repellent, was being found at extremely low levels throughout the environment and the human population. Since that time, environmental scientists and toxicologists have begun paying much more attention to PFOS, its sister compound perfluorooctanoic acid (PFOA; known for its use in DuPont’s Teflon products), and other members of the family of perfluoroalkyl acids (PFAAs). As more tests have been conducted, the research has revealed that laboratory animals respond in vastly different ways to PFAAs and related compounds, which can make it difficult to pinpoint the mechanisms underlying the responses. However, toxicologists are making headway in their understanding of these compounds, an important fact in light of new research suggesting that the levels being found in both people and animals may have an impact on their health.

The tremendous variation in the speed with which humans and laboratory animals can eliminate PFOA is one example of why understanding how the compounds are processed in the body poses such a formidable challenge. “You go from hours for the female rat, to days for the male rat, to months for the monkey, to almost four years in humans,” explains Jennifer Seed, a branch chief with the EPA Office of Pollution Prevention and Toxics.

“We truly don’t understand what are the biological events that drive this difference,” says Christopher Lau, a lead research biologist with the EPA National Health and Environmental Effects Research Laboratory (NHEERL). “Are there binding protein differences? Do humans have a different set of transporters that is not the same as in animals?” Lau terms these gaps in understanding “a black hole.”

These gaps render the toxicologist’s goal of extrapolating from one species to another “a very complex state of affairs,” as Seed puts it. For this reason, deciphering the human risk posed by exposure to PFAAs is a major challenge, Lau says. “We need to go to the next level to identify the underlying events that drive the adverse effects,” he says.

## Anatomy of a PFAA

The compounds used in commercial perfluorinated formulations are sometimes identified by the number of carbon atoms they contain. In general, the longer the carbon chain length, the more the PFAA persists in the body, according to Naomi Kudo, an associate professor of toxicology and applied pharmacology at Josai University in Japan. For example, perfluorobutane sulfonate (PFBS), which has 4 carbons, is eliminated in a little over 1 month in humans, on average, while PFOA and PFOS (so-called C8 compounds with 8 carbons each) are eliminated in 3.8 and 5.4 years, respectively. Perfluorohexane sulfonate (PFHxS), with 6 carbons, is an exception to the rule; it is eliminated in 8.5 years.

3M no longer manufactures PFOS, and the compound is now used only in relatively small quantities for applications for which there is no acceptable substitute, such as in semiconductor manufacturing. All eight of the companies currently using PFOA—Arkema, Asahi, Ciba, Clariant, Daikin, DuPont, 3M/Dyneon, and Solvay Solexis—have agreed to reduce PFOA releases and levels in products by 95% by 2010 and to eliminate their use by 2015.

The new compounds being introduced to replace PFOA and PFOS fall into three general groups: the perfluoroalkyl sulfonates (a group that includes PFOS), the perfluoroalkyl carboxylates (including PFOA), and the fluorotelomer alcohols, which are used to produce perfluorinated surfactants and polymers for products including hair care products, paper products used in direct contact with food, rug cleaners, and lubricants for bicycles, garden tools, and zippers, according to the nonprofit Environmental Working Group. 3M is building its new PFAA products around compounds containing fewer carbons, including PFBS, because of their shorter half-lives in humans, says John Butenhoff, a corporate scientist in toxicology for 3M’s Medical Department.

But some of the new replacement compounds may pose problems of their own. More than 20 different such compounds were discussed at a 14–16 February 2007 meeting of the Society of Toxicology (SOT) on the toxicokinetics and mode of action of PFAAs and related chemistries. For example, fluorotelomer alcohols are emerging as the main remaining source of PFOA in the environment. These and other “residual” compounds can be transformed into PFOA or PFOS as the result of metabolism or environmental biodegradation. In a presentation at the SOT conference, Butenhoff noted that 1% of the total dose of 8-2 fluorotelomer alcohol given to laboratory rats is metabolized to PFOA. Similarly, other researchers have observed that *N*-ethyl-*N*-(2-hydroxy-ethyl)perfluorooctoanesulfonamide, a constituent of coatings used on paper and cardboard, can be transformed into PFOS in the environment. It also may produce PFOA in the atmosphere.

Other PFAAs being detected in the environment are also receiving more attention. The CDC detected not just PFOS and PFOA but also PFHxS, perfluorononanoic acid, and perfluorooctane sulfonamide in every U.S. human blood sample from the 1999–2000 National Health and Nutrition Examination Survey (NHANES) that was analyzed for PFAAs, according to Antonia Calafat, a lead research chemist at the CDC’s National Center for Environmental Health. In a paper in the 1 April 2007 issue of *Environmental Science & Technology*, CDC researchers also reported finding two compounds used in surfactants and coatings on fabric, paper, and upholstery—2-(*N*-ethyl-perfluorooctane sulfonamido) acetic acid and 2-(*N*-methyl-perfluorooctane sulfonamido) acetic acid—in more than 90% of the samples, she says. Similarly, perfluorobutyrate (a 4-carbon compound) has been detected in surface water and public and private wells. Lau adds that PFOS and PFOA have been found at locales near PFAA production plants and waste disposal facilities.

## Polar Bears, Pandas, and People

Although a growing body of research is focused on other PFAAs, PFOA and PFOS have been the subject of the lion’s share of study to date. Both compounds are found throughout the environment—from polar bears living in Greenland, to giant pandas in China, to albatrosses on the Midway Atoll in the middle of the Pacific Ocean. The compounds are also widely dispersed in surface waters, according to 3M.

At the SOT meeting in February, researchers from the New Jersey Department of Environmental Protection (NJDEP) reported detecting PFOA in drinking water samples from 78% of 23 treatment plants sampled and PFOS in samples from 57% of the plants. The finding prompted the NJDEP to recommend in February 2007 that the state move toward regulating PFOA in water. Currently New Jersey recommends that the concentration of PFOA in drinking water be less than 0.04 ppb.

This value is significantly lower than the Site-Specific Action Level of 0.5 ppb developed by the U.S. EPA as part of a consent order in 2006 with DuPont for drinking water in Ohio and West Virginia impacted by DuPont’s Washington Works facility in West Virginia. (This action level applies only to the DuPont–West Virginia settlement; there is no federal standard for PFOA in drinking water.) The highly publicized C8 Study conducted by Edward Emmett and colleagues the University of Pennsylvania has examined drinking water exposures to PFOA among Ohio and West Virginia residents living near the Washington Works plant. At the start of the study, the PFOA concentrations in the blood serum of residents in Little Hocking, Ohio, were up to 89 times higher than the U.S. average, according to a report by Emmett in the August 2006 *Journal of Occupational and Environmental Medicine*. At press time, investigators on the study expected any day to release results of whether use of bottled drinking water had reduced these concentrations.

The NJDEP findings “suggest that PFOA is commonly present in public water systems not known to be specifically contaminated by a point source,” says Gloria Post, a toxicologist with the department. Additionally, Emmett’s *Journal of Occupational and Environmental Medicine* paper indicates that even low concentrations of PFOA in drinking water may significantly contribute to levels found in the general population.

People can also be exposed to PFOA and PFOS due to poor disposal practices. In Germany, industrial waste contaminated with high concentrations of PFAAs was mixed with soil by a recycling company. Although the amended soil was later declared illegal as a “soil improver,” it was nonetheless used by farmers in the Arnsberg agricultural area in the country’s North Rhine–Westfalia state, according to Martin Kraft of the state’s Ministry of the Environment and Conservation, Agriculture, and Consumer Protection. When Kraft and his colleagues analyzed how PFOA and PFOS had spread through the environment, they found concentrations of the two compounds together reached 148 ppb in surface waters and 0.6 ppb in drinking water, according to a poster he presented at the SOT meeting. The concentrations in edible fish including trout, chub, and eel reached as high as 1.2 ppm, with a median of 133 ppb. In comparison, similar fish from unpolluted waters contained an average of 4 ppb.

PFOA was the predominant compound detected in serum from the area’s people, whose average serum levels of the compound were 6 to 8 times higher than an unexposed region of the country, Kraft says. In a German-language government document published 15 March 2007, Kraft and his colleagues reported that the average serum PFOA concentration in Arnsberg children was 22.1 ppb, in women it was 24.9 ppb, and in men it was 27.4 ppb.

For the U.S. population, CDC researchers analyzed NHANES samples collected in 1999–2000 to produce the first nationally representative survey of PFAAs, and these data are meant to serve as a baseline, Calafat says. The average concentration of PFOS in the 1,562 serum samples collected from people aged 12 years and older was 30.4 ppb, whereas the average concentration of PFOA was 5.2 ppb. The levels in men were slightly higher, on average, than those in women, and the people with the highest levels of the compounds also were the most educated.

PFOA and PFOS have also been detected in human breast milk and babies’ blood. A Swedish study in the February 2007 issue of *EHP* calculated that the total amount of PFAAs transferred to breastfeeding infants was approximately 200 ng/day.

3M researchers have collected some evidence that the company’s decision to phase out production of materials including PFOS and greatly reduce its use of PFOA by the end of 2002 was already beginning to affect levels of the compounds three years later. In a pilot study published in the May 2007 issue of *Chemosphere*, 3M researchers compared concentrations of PFOA and PFOS in plasma samples taken from 40 American Red Cross donors in the Minneapolis–St. Paul area in 2005 with 100 samples taken five years earlier from the same general population. They found that the average concentrations of both PFOA and PFOS in the donor samples dropped by more than 50% over that five-year period, says Geary Olsen, a staff scientist with 3M’s Corporate Occupational Medicine Department.

The information gleaned from 3M’s pilot study is not directly comparable to the PFAA data from the 1999–2000 NHANES because it is a random sample and not statistically representative of the U.S. population, Olsen acknowledges. However, he points out that a study of concentrations of PFOA and PFOS in American Red Cross donations in six cities in 2000, which was published in the December 2003 issue of *EHP*, produced numbers that were nearly identical to what the CDC has reported for the same time frame. 3M has just completed analyzing the samples from a follow-up study conducted in 2006 that involves samples from the same six cities and expects to submit them for publication later this year. The company hopes these new data will validate the drops in PFAA concentrations seen in the pilot study, Olsen adds.

## Health Effects in Animal Studies

In animal studies, toxicologists have seen that high doses of both PFOS and PFOA cause cancer, physical development delays, endocrine disruption, and neonatal mortality. This last effect is arguably the most dramatic result of laboratory animal tests with PFOS and PFOA. “Animals are born, they look quite healthy and pink, and then they die quite rapidly,” Seed says. Other studies show that the compounds can impact growth and development and disrupt the body’s hormone and immune systems.

In older animals, toxicological studies have shown that the compounds cause liver and pancreatic tumors. A number of studies have demonstrated the ability of both PFOS and PFOA to bind to peroxisome proliferator–activated receptors (PPARs), a class of receptors associated with carcinogenesis. In addition to being investigated as a cause of the cancers seen in laboratory animals, PPAR activation is believed to affect fetal growth and immune function.

Much of the research conducted to date has focused on the ability of PFAAs to act as PPAR agonists by triggering a response to a key receptor isoform, PPAR-α. New research is beginning to show that the compounds affect other aspects of the body’s biochemistry, Seed says; in fact, both PFOA and PFOS may have multiple mechanisms of action.

By working with mice genetically engineered not to contain PPAR-α, Barbara Abbott, a research biologist at NHEERL, implicated that isoform in the neonatal mortality caused by PFOA exposure. Because PFOA is a fairly potent PPAR-α agonist (much more so than PFOS), the work suggests that different mechanisms are responsible for the PFOS-induced neonatal mortality seen in animals. “Both PFOS and PFOA cause neonatal mortality, and it is tempting to suggest that they have the same mode of action, but in reality, they may not at all,” points out John Rogers, chief of the Developmental Biology Branch of the NHEERL Reproductive Toxicology Division.

At the SOT meeting, Kudo presented research showing that the way male rats process low doses of PFOA differs from how they process high doses. These studies show that the compound is preferentially taken up by the liver and is more likely to be excreted from the liver into the bile only at higher doses. Kudo’s research may help account for why 3M plant workers exposed to low doses tended to retain the compound in their bodies for such long periods, while laboratory rats exposed to high doses quickly removed the compound from their bodies, she says. The work may also explain why female rats can rid their bodies of PFOA so much more quickly than males can, according to Butenhoff.

Scientists have also made some progress in understanding how PFOA and PFOS cause neonatal mortality in laboratory mice. Researchers at the EPA have determined that newborn mice treated with these substances that appeared to be unable to breathe were biochemically mature and genetically normal, Rogers said at the SOT meeting. The latest hypothesis is that PFOS may impede the function of the endogenous pulmonary surfactant needed to inflate the lungs, he says.

## The Human Health Impact

What does all this mean for human health? To provide a more useful context for comparing human data with the insights derived from animal studies, researchers working with laboratory animals should be determining the concentrations of PFAAs in the bodies of their test subjects, rather than simply reporting the administered dose, stresses Melvin Andersen, director of the Computational Biology Division of The Hamner Institutes For Health Research.

Although most of the studies showing adverse effects in laboratory animals involved much higher levels of PFOS and PFOA than are actually being seen in humans and other animals, as-yet unpublished research conducted at environmentally relevant concentrations suggests that exposure at such levels may have an effect on humans.

Researchers at The Johns Hopkins University found PFOA in 100% and PFOS in 99% of 297 serum samples collected in 2004 and 2005 from the umbilical cords of children born in Baltimore, according to Lynn Goldman, a pediatrician and epidemiologist at the Bloomberg School of Public Health. Overall, the levels were lower than in adults, but the highest concentration of PFOS detected was 34.8 ppb, says Goldman, who stresses that these unpublished results need to be confirmed. The source of the PFOA and PFOS in the infants’ blood was unclear, though research published online 12 January 2007 in the *Archives of Occupational and Environmental Health* suggests that transplacental transfer may account for it.

In addition to revealing a statistically significant correlation between infants born with higher levels of PFOS and PFOA and decreased birth weight and head circumference, the Johns Hopkins study unearthed a correlation between the compounds and the scores the babies earned on the ponderal index, which measures fetal body mass and can serve as a rough approximation of nutritional status. “The lower the ponderal index, the higher the [cord serum] PFOS and PFOA [concentrations],” Goldman says. Other studies have suggested that low birth weight may be a risk factor for obesity, diabetes, and cardiovascular diseases later on.

The Johns Hopkins researchers also correlated the babies’ PFOA (but not PFOS) concentrations with their total circulating thyroxine levels, Goldman says. Higher concentrations of PFOA and PFOS were linked with longer gestational periods, as well. This raises the question of whether these compounds are transported more readily later in pregnancy, or accumulate with the fetus during pregnancy, says Goldman.

Unlike the CDC study, the Johns Hopkins research did not find any correlation between the socioeconomic status of the parents and the children’s blood PFOA and PFOS concentrations, which Goldman says is “quite remarkable.” Because the babies were born into families from a wide socioeconomic range, and because other research points to consumer products as the source of the compounds, Goldman says the new study suggests that if consumer products are the source, “they are the ones everyone in our [study] group is using.”

Given the association in Goldman’s research of higher levels of PFOS and PFOA with lower ponderal index scores, some researchers wonder if this finding could tie in with new evidence connecting high levels of PFOA in rodent pups to obesity later in life. The research to date shows that the offspring of exposed pregnant mice have a dose-related increase in obesity, Rogers explains. “By the time they’re obese,” he says, “they have very little remaining PFOA and their liver is back to normal size.”

One hypothesis for why this is happening is that PFOA could be acting as a hypolipidemic agent in increasing fatty acid metabolism, according to Rogers. In other words, the PFOA treatment is “essentially asserting an [undernourished] environment *in utero*.”

“We know that these compounds affect fatty acid metabolism,” Seed says. “Maybe something is happening in the developing organism that is interfering with the program of energy metabolism.”

Rogers says this fits with what is known as fetal programming syndrome in human infants, in which children who experience a prenatal environment chronically short of nutrients and are then reared with an abundance of food are more likely to become overweight. “Whatever is happening that mediates its effects on lipid metabolism, whether through PPAR-α or otherwise, could be very important,” he says. “We know very little about what’s going with the fetus in terms of metabolic programming. The environment, in a very critical period of development, might affect metabolism or shift metabolism for a lifetime.”

However, Lau points out that PFOA is but one of a number of environmental contaminants that are being linked to adult obesity. Follow-up research is in order to more carefully pinpoint the events that lead to obesity, perhaps by looking at gene expression or protein markers for adipogenesis earlier in test animals’ lifetimes.

More research is needed, agrees Suzanne Fenton, the EPA research biologist who conducted most of the research linking prenatal PFOA exposure in mice with adult obesity. She says the latest data suggest this effect is being seen at dosages below 1 mg of PFOA per kg of body weight (the actual amount of PFOA in the animals’ blood was not determined). Her studies also revealed PFAA-induced abnormalities in other mouse tissues, including the ovaries, mammary glands, and spleen.

## Immunotoxicity: The Case of Atlantic Dolphins

In light of research suggesting that PFOA and PFOS both cause potent suppression of the adaptive immune system, in 2006 the EPA Science Advisory Board called for immunotoxicity to be the subject of more study. The EPA is currently conducting such studies and has replicated findings showing that PFOA suppresses the primary immune response, says Robert Luebke, a research biologist with the NHEERL Immunotoxicology Branch. The researchers are looking for PPAR-α activity, but there are some indications that something else may be going on, he says. He and his colleagues have noticed that the adrenal glands of treated mice are somewhat enlarged, which fits with reports that corticosteroid levels rise in PFOA-treated animals.

The first research to suggest that the levels of PFAAs being detected in wild animals could be impacting their immune systems involved bottlenose dolphins believed to have “the highest [PFOS levels] ever reported in any wildlife species,” according to Margie Peden-Adams, an assistant professor at the Medical University of South Carolina Department of Pediatrics and Marine Biomedicine and Environmental Science Center. At the SOT meeting, she presented a poster discussing her work with an international team that analyzed blood samples collected from 89 dolphins living near Charleston, South Carolina, and Indian River Lagoon, Florida. The animals harbored concentrations of PFOA that were approximately twice the levels that the CDC found in U.S. citizens, but their average levels of PFOS were 20 to 40 times higher, according to an analysis published by a team of University of Guelph researchers in the 1 October 2006 edition of *Environmental Science & Technology*.

In conjunction with collaborators at Clemson University and the Mystic Aquarium, Peden-Adams helped develop a suite of assays to test immune function in the bottlenose dolphin. “We did not find overwhelming suppression [associated with PFAAs],” she says. For example, the researchers observed no alterations in T-cell proliferation or NK-cell activity. However, lysozyme activity was suppressed, B-cell proliferation was stimulated, and numbers of various lymphocytes increased. “The immune system is very compensatory, and often when one thing is suppressed, another thing may be increased,” she says.

“It is important to note that any deviation on the continuum of possible immune effects from normal homeostasis is considered an alteration,” Peden-Adams stresses. “Suppressed immune function can lead to increased vulnerability to pathogens, but enhanced immune function can be detrimental as well, leading to hypersensitivity reactions, allergy, and autoimmune reactions.”

For comparison, Peden-Adams and her colleagues dosed B6C3F1 mice with PFOS at concentrations comparable to those found in the dolphins. “The effects on antibody production seen in the mice are what would be expected based on studies with PFOA and PFDA [perfluorodecanoic acid] and . . . occurred at environmentally relevant exposure levels as compared to control animals,” she says. This new research is noteworthy because no studies to date have determined the immune effects of PFOS, and “no other laboratories we are aware of are assessing [these effects],” Peden-Adams says.

## Next Up for PFAA Research

The toxicological research conducted to date wtih PFAAs shows “profound changes in the biochemistry of [test] animals,” Andersen says. “I believe that enough work has been done to have a hypothesis that most of the responses are coming from some receptor-mediated processes.” Andersen therefore proposes that it makes sense for the research community to move forward with low-dose studies that attempt to look for genetic or genomic changes associated with effects such as immunotoxicity and reproductive toxicity. Rogers agrees, although he points out that such low-dose studies can be very difficult to conduct because the effects are more subtle, and carrying them out can involve the use of hundreds of test animals.

“Doing more human population studies is another approach,” says Goldman, who adds that closer collaboration between toxicologists and epidemiologists would aid such an effort immensely. In animal studies, toxicologists can “look directly at biomarkers and molecular changes in the brain, kidney, and the liver—anywhere they wish—whereas in human studies, we are limited by what is available without creating an excessive burden on research subjects,” she says.

“If we’re going to bring the fields closer together, we need to have human epidemiological research that is focusing more on mechanisms,” Goldman adds. For example, she says that environmental health research would be much more relevant to epidemiologists if toxicologists would work toward identifying biomarkers in human serum that are indicative of risk. “If we’re interested in what the effects are in humans, then one of the things I think we need to do better is to begin thinking about modes of action in people as well as toxicology,” she concludes.

## Figures and Tables

**Figure:**
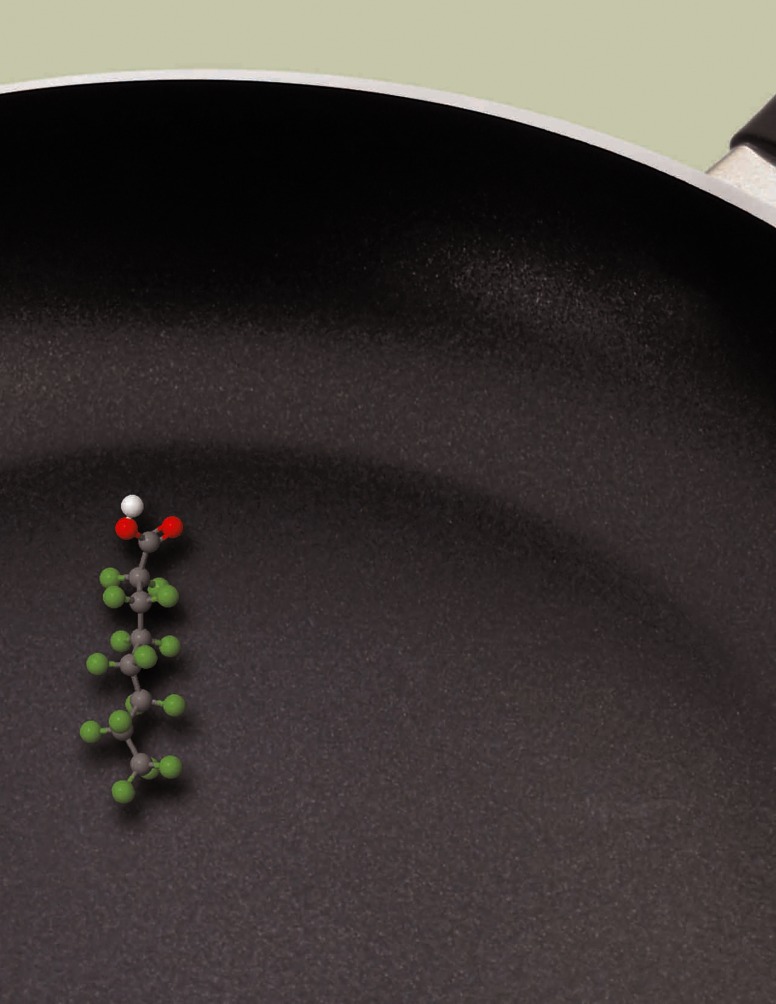


**Figure:**
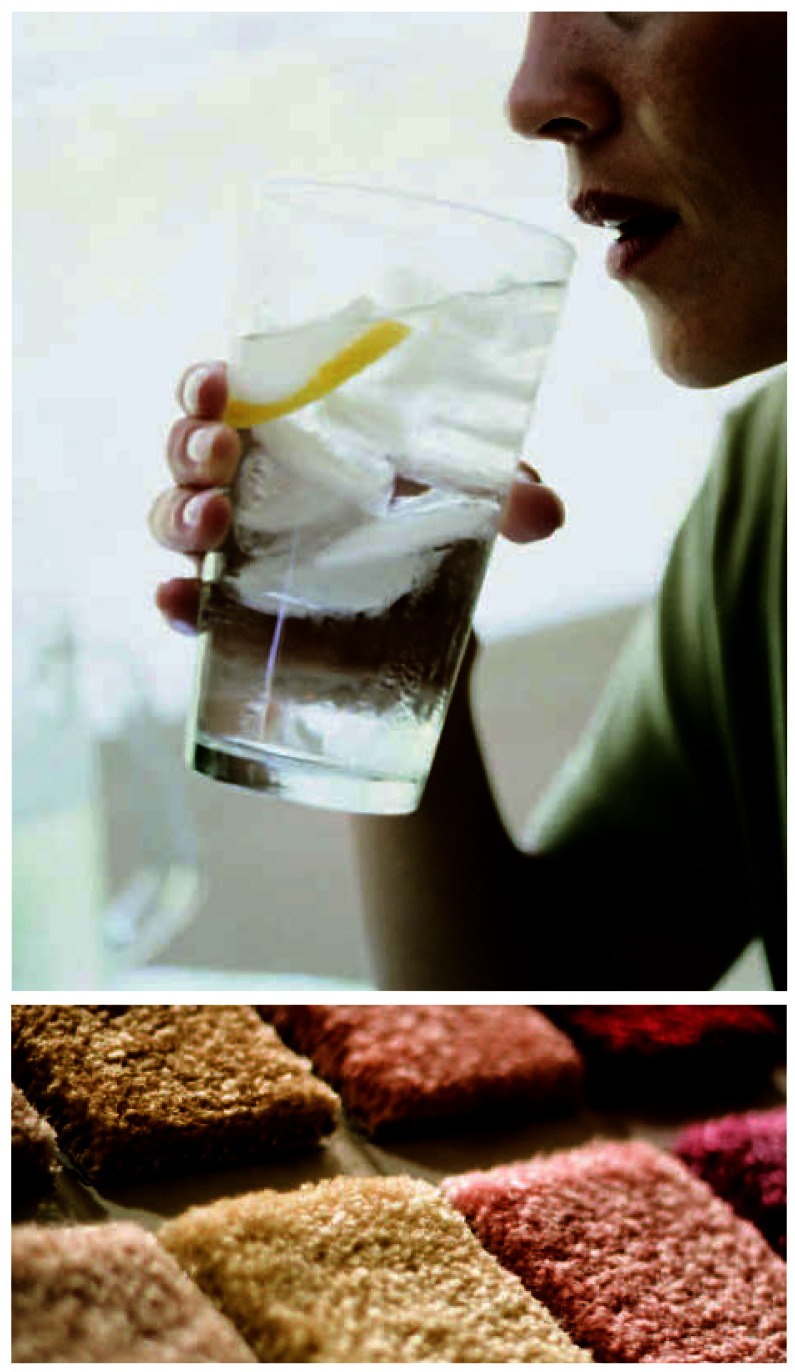
Human exposure to PFAAs comes through myriad sources including contaminated drinking water and household products treated with stain or water repellants.

**Figure:**
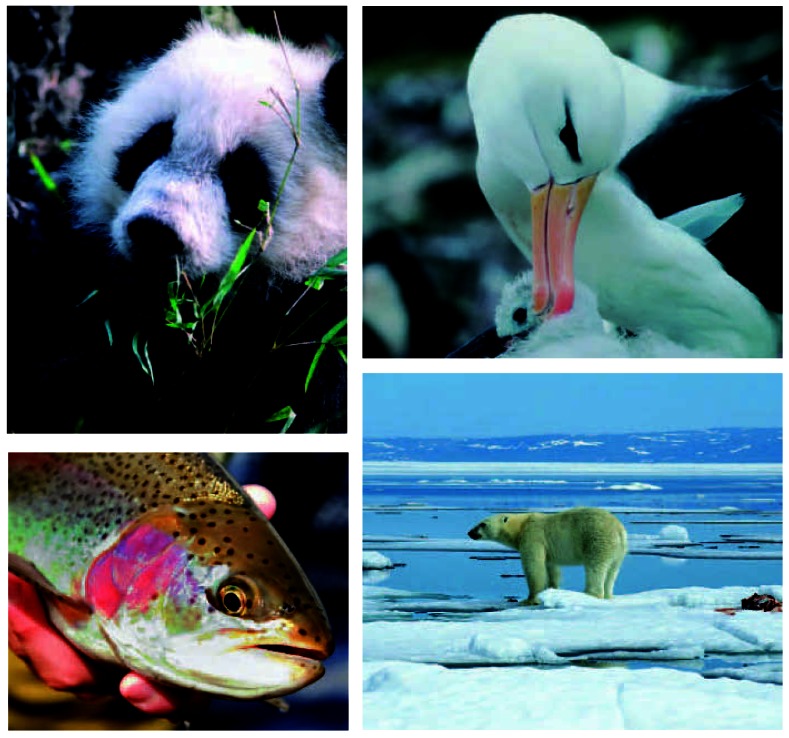
PFAAs are ubiquitous in the environment, found on every continent in the world, in numerous mammal, fish, and bird species.

**Figure:**
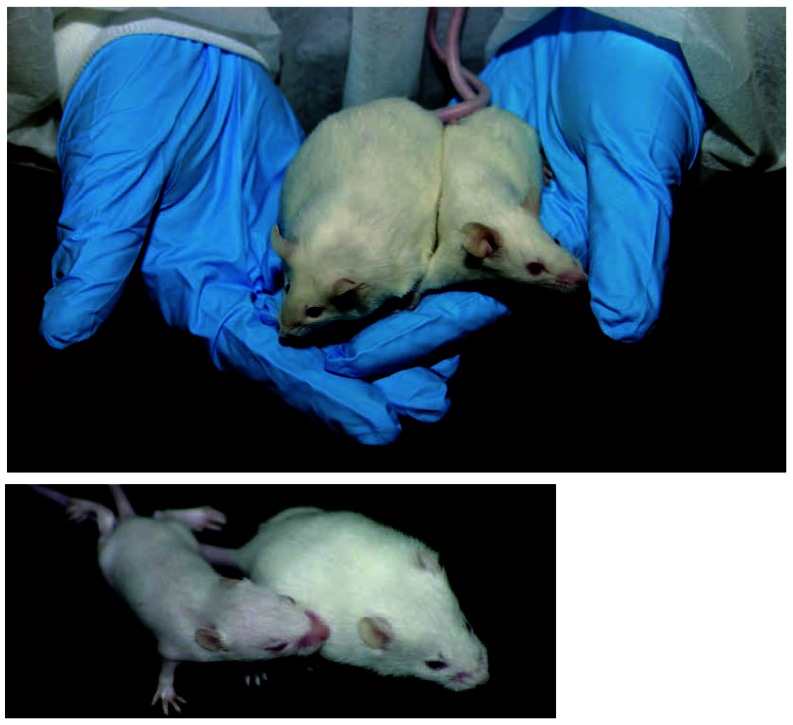
Laboratory mice exposed prenatally to PFOS and PFOA develop more slowly and suffer a higher rate of neonatal mortality than nonexposed mice (left). Once exposed mice reach adulthood, however, they are more likely to become obese (above).

**Figure:**
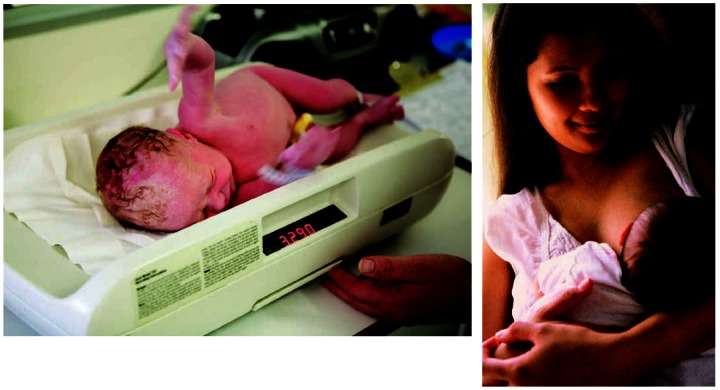
Prenatal exposure to PFOS and PFOA can affect body weight and head circumference in human infants. Postnatal exposures, as through breastfeeding, have unknown effects.

**Figure:**
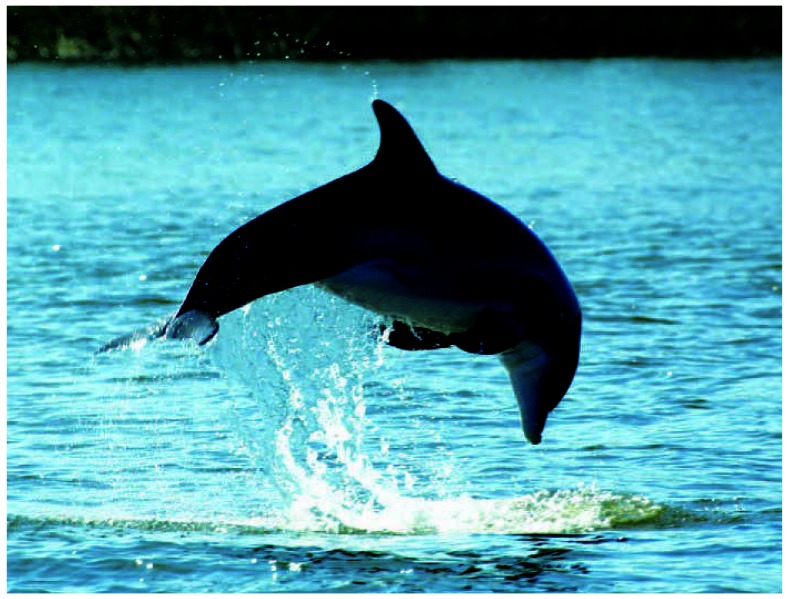
Studies of bottlenose dolphins with some of the highest levels of PFOS reported in wild animals indicate that the chemical may affect immune function.

