# Erratum

**Published:** 2007-07

In the May Focus article, “Perfluoroalkyl Acids: What Is the Evidence Telling Us?” [ Environ Health Perspect 115:A250–A256 (2007)], the caption on page A254 should have read “Once PFOA-exposed mice reach adulthood, however, they are more likely to become obese (above)”; PFOS (perfluorooctanyl sulfonate) exposure *in utero* has not been linked to later obesity in laboratory animals. In addition, the caption on page A255 may be interpreted as implying causality between prenatal exposure to PFOS and PFOA (perfluorooctanoic acid) and altered body weight and head circumference in human infants. This was not *EHP*’s intention. In fact, although an association has been shown, causality has not been established. On the same page, the journal identified as *Archives of Occupational and Environmental Health* is actually *International Archives of Occupational and Environmental Health*.

*EHP* regrets the errors.

